# Gene Expression Changes in Venous Segment of Overflow Arteriovenous Fistula

**DOI:** 10.1155/2013/980923

**Published:** 2013-04-27

**Authors:** Yasuhiro Hashimoto, Akiko Okamoto, Hisao Saitoh, Shingo Hatakeyama, Takahiro Yoneyama, Takuya Koie, Chikara Ohyama

**Affiliations:** ^1^Oyokyo Kidney Research Institute, Hirosaki Hospital, Hirosaki 036-8243, Japan; ^2^Department of Urology, Hirosaki University Graduate School of Medicine, 5 Zaifucho, Hirosaki, Aomori 036-8562, Japan

## Abstract

*Aim*. The objective of this study was to characterize coordinated molecular changes in the structure and composition of the walls of venous segments of arteriovenous (AV) fistulas evoked by overflow. *Methods*. Venous tissue samples were collected from 6 hemodialysis patients with AV fistulas exposed to overflow and from the normal cephalic veins of 4 other hemodialysis patients. Total RNA was extracted from the venous tissue samples, and gene expression between the 2 groups was compared using Whole Human Genome DNA microarray 44 K. Microarray data were analyzed by GeneSpring GX software and Ingenuity Pathway Analysis. *Results*. The cDNA microarray analysis identified 397 upregulated genes and 456 downregulated genes. Gene ontology analysis with GeneSpring GX software revealed that biological developmental processes and glycosaminoglycan binding were the most upregulated. In addition, most upregulation occurred extracellularly. In the pathway analysis, the TGF beta signaling pathway, cytokines and inflammatory response pathway, hypertrophy model, and the myometrial relaxation and contraction pathway were significantly upregulated compared with the control cephalic vein. *Conclusion*. Combining microarray results and pathway information available via the Internet provided biological insight into the structure and composition of the venous wall of overflow AV fistulas.

## 1. Introduction

Arteriovenous (AV) fistulas are very useful for determining optimal blood flow for dialysis, but AV fistulas exposed to overflow are thought to increase cardiac output and cause high-output cardiac failure [1, 2].

Measurement of blood flow via an internal shunt was first developed by Krivitski et al., and the monitoring of blood flow via a shunt has since become widespread [3]. We use this technique to monitor the blood flow of AV fistulas at our hospital and correct overflow AV fistulas with surgery.

It is thought that the outflow vein of overflow AV fistulas bears a heavy load: as the vein is exposed to increased arterial flow, the wall dilates, triggering a vascular remodeling process. However, the molecular mechanisms by which the outflow vein is remodeled into a mature fistula remain unclear. By investigating venous remodeling in overflow AV fistulas, candidate genes important to the remodeling process can be discovered and their functional significance investigated. Thus, the identification of relevant genes involved in this process should provide insight into AV fistula maturation.

In this study, we performed a cDNA microarray analysis and compared segments of the venous walls of overflow AV fistulas from 6 hemodialysis patients with the normal cephalic veins of 4 other hemodialysis patients to determine whether there was any difference in their gene expression patterns. 

## 2. Study Population

**Figure 1:**
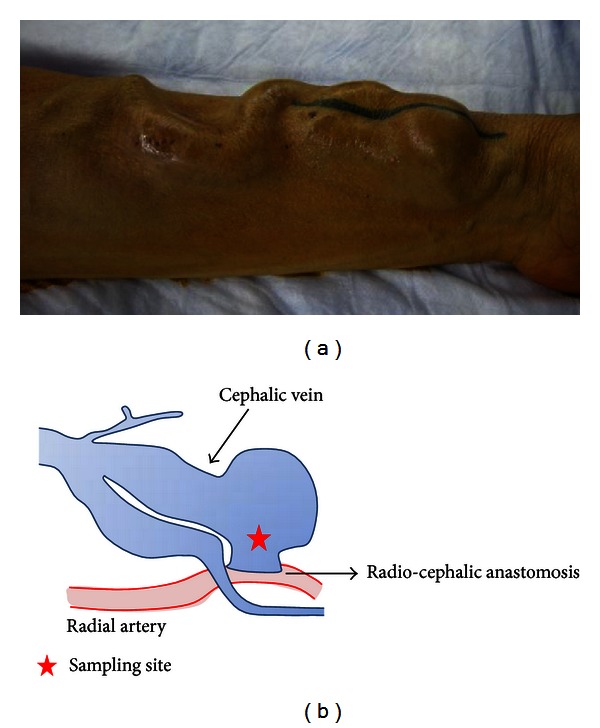
(a) Photograph of overflow AV fistula. (b) Schematic of overflow AV fistula.

From June 2009 to September 2010, 548 patients underwent hemodialysis at the Oyokyo Kidney Research Institute in Hirosaki, Japan. During that period, 10 patients underwent surgical ligation to correct an overflow AV fistula. When the operation was performed, we retained a sample of the wall of the overflow AV fistula (Figure 1). The AV fistula specimens were resected from the wall of the vein close to the AV fistula anastomosis. The study was approved by the Bioethics Committee of Oyokyo Kidney Research Institute, and all patients provided their informed consent to the procedure prior to it being performed. 

## 3. Inclusion Criteria

The inclusion criteria were as follows: (1) blood access flow greater than 2.0 L/min measured by the color Doppler ultrasound (2) an AV fistula in the lower arm with a distal radio-cephalic anastomosis. In total, 6 patients had overflow AV fistulas that met these criteria. The backgrounds of these patients are detailed in Table 1. We also obtained tissue samples from the lower arm distal cephalic veins of 4 new hemodialysis patients and used these as a control. 

## 4. Methods

As noted above, venous tissues were resected from a venous segment of an overflow AV fistula from 6 patients and from a normal cephalic vein from 4 other patients. The surgical specimens were immediately placed in test tubes containing RNAlater (see below for details).

Total RNA was extracted from the venous tissue samples, and gene expression between the 2 groups was compared using Whole Human Genome DNA microarray 44 K (Agilent Technologies, Santa Clara, California). The microarray data were analyzed with GeneSpring GX software and Ingenuity Pathway Analysis. 

## 5. RNA Isolation

Surgical specimens were 0.5 cm or smaller in size and were initially stored in RNA later (Ambion, Austin, TX) overnight at 4 ± 3°C then at –80°C until RNA extraction. Total RNA was extracted using TRIzol reagent (Invitrogen, Carlsbad, CA) according to the manufacturer's instructions. The total RNA was further purified using the Qiagen RNeasy Mini Kit (Qiagen, Valencia, CA) and then extracted. The quantity and quality of the RNA were determined using a Nanodrop ND-1000 spectrophotometer (Thermo Fisher Scientific Inc., Waltham, MA) and an Agilent Bioanalyzer (Agilent Technologies, Palo Alto, CA). 

## 6. cRNA Amplification and Labeling

Total RNA was amplified and labeled with Cyanine 3 (Cy3) as instructed by the manufacturer of the Agilent Low Input Quick Amp Labeling Kit, one-color (Agilent Technologies, Palo Alto, CA). Briefly, 100 ng of total RNA was reverse transcribed to double-strand cDNA using a poly dT-T7 promoter primer. The primer, template RNA, and quality-control transcripts of known concentration and quality were then denatured at 65°C for 10 min and incubated for 2 hours at 40°C with 5X First-Strand Buffer, 0.1 M DTT, 10 mM dNTP mix, and Affinity Script RNase Block Mix. The Affinity Script enzyme was inactivated at 70°C for 15 min. The resulting cDNA products were then used as templates for in vitro transcription to generate fluorescent cRNA. They were mixed with a transcription master mix in the presence of T7 RNA polymerase and Cy3-labeled CTP and incubated at 40°C for 2 hours. Labeled cRNAs were purified using Qiagen's RNeasy Mini spin columns and eluted in 30 *μ*L of nuclease-free water. After amplification and labeling, cRNA quantity and cyanine incorporation were determined using a Nanodrop ND-1000 spectrophotometer and an Agilent Bioanalyzer.

## 7. Sample Hybridization

For each hybridization, 1.65 *μ*g of Cy3-labeled cRNA was fragmented and hybridized onto an Agilent Human GE 4x44K v2 Microarray (Design ID: 026652) for 17 hours at 65°C. After washing, the microarrays were scanned using an Agilent DNA microarray scanner.

## 8. Microarray Data Analysis

The intensity values of each scanned feature were quantified using Agilent feature extraction software (version 10.7.3.1), which performs background subtractions. We only used features flagged as having no errors (present flags) and excluded features that were not positive, not significant, not uniform, not above background levels, saturated, or population outliers (marginal and absent flags). Normalization was performed using Agilent GeneSpring GX version 11.0.2. (per chip: normalization to the 75 percentile shift; per gene: normalization to median across all samples). There are 34,127 probes in total on the Agilent Human GE 4x44K v2 Microarray (Design ID: 026652), excluding control probes. The microarray data were submitted to NCBI GEO (http://www.ncbi.nlm.nih.gov/geo/), sample number [GSE39488].

The altered transcripts were quantified using a comparative method. We applied a *P* value < 0.05 combined with a >2-fold change in normalized intensity to identify genes with significantly different expression patterns.

## 9. Gene Ontology Analysis and Pathway Analysis

The gene ontology analysis was performed using Agilent Technologies GeneSpring GX software (11.0.2). Pathway analysis was performed with GenMAPP 2.1 (http://www.genmapp.org/).

## 10. Results

The cDNA microarray analysis revealed that 397 genes were upregulated and 456 were downregulated (Tables 2 and 3).

The gene ontology analysis revealed that biological developmental processes and glycosaminoglycan binding were the most upregulated. In addition, most upregulation occurred extracellularly (Tables 4, 5, and 6).

The pathway analysis revealed that the TGF beta signaling pathway, cytokines and inflammatory response pathway, hypertrophy model, and the myometrial relaxation and contraction pathway were upregulated (Table 7).

## 11. Discussion

AV fistulas are very useful for determining the optimal blood flow for hemodialysis since satisfactory blood access flow is necessary for adequate hemodialysis. When stenotic lesions occur within the vascular system and blood flow is insufficient, a percutaneous transluminal angioplasty or some other intervention is performed. However, overflow AV fistulas increase cardiac output and cause high-output cardiac failure [1].

In the 2005 Japanese Society for Dialysis Therapy Guidelines for Vascular Access Construction and Repair for Chronic Hemodialysis, vascular access flow is said to lead to heart failure when the blood access flow is greater than 1.0–1.5 L/min or when the vascular access flow/cardiac output ratio is >20% [1]. If the vascular access flow is clearly responsible for a decline in cardiac function, then it is necessary to intentionally constrict or occlude the vascular access [1]. Surveillance of blood flow in internal shunts by the Doppler echocardiography has become widespread and overflow AV fistulas are now actively treated. Several recent studies have noted the importance of histological changes in AV fistulas [4, 5]. 

Microarrays of vascular access have been reported in experimental animal models, but there have been no such analyses in humans [6]. In the present study, venous tissue samples were resected from overflow AV fistulas from 6 hemodialysis patients and from the normal cephalic veins of 4 other hemodialysis patients, and their gene expression patterns were compared.

It is interesting to note that zinc finger-containing transcription factors such as egr1, egr2, and egr3 and immediate early genes such as fos and jun, were found to be remarkably upregulated in the present study; egr1, egr 2, and egr 3 have been implicated in the proliferation and differentiation of many cell types [7, 8], and fos and jun have been linked to the regulation of angiogenesis [9]. Moreover, egr-1, c-jun, and c-fos have been linked to the regulation of free radical scavenging enzymes [10–13]. We also observed the upregulation of free radical scavenging enzyme activity in the walls of the overflow AV fistulas, which may reflect chronic reactive oxygen species formation in overflow AV fistulas. 

The pathway analysis indicated that the TGF beta signaling pathway and cytokines and inflammatory response pathway were upregulated. This suggests that overflow AV fistulas may be implicated in chronic inflammation in hemodialysis patients. 

Malnutrition, inflammation, and atherosclerosis (MIA syndrome) are common in end-stage renal disease (ESRD) patients, and inflammation has been identified as playing a key role in atherosclerotic cardiovascular disease. Proinflammatory cytokines are pivotal to the inflammation that is, associated with malnutrition and atherosclerosis in ESRD [14]. Our findings suggest that overflow AV fistulas may be implicated in MIA syndrome.

## 12. Conclusion

Combining microarray results and pathway information available via the Internet provided biological insight into molecular changes in the venous walls of overflow AV fistulas. Despite the small sample size, our study findings suggest that overflow AV fistulas may be implicated in chronic inflammation in hemodialysis patients.

## Figures and Tables

**Table 1:** Patient characteristics.

Over flow AVF	Age	Gender	Cause of CRF	Patency period of AV fistula (months)	Blood flow (mL/min)
1	48	M	CGN	56	3790
2	83	F	CGN	93	2760
3	57	M	CGN	19	3280
4	46	M	CGN	22	2710
5	75	M	CGN	104	3520
6	57	F	IgA	88	2340

Control					
1	67	M	CGN	(−)	(−)
2	68	F	CGN	(−)	(−)
3	56	M	CGN	(−)	(−)
4	80	F	CGN	(−)	(−)

**Table 2:** Genes significantly upregulated in the remodeled vein compared to the control vein (top 30).

Probe name	*P* value	FCAbsolute	Gene symbol
A_23_P106194	0.045653246	38.85	FOS
A_23_P429998	0.021514166	27.22	FOSB
A_24_P33895	0.001204797	25.26	ATF3
A_23_P46936	1.36*E* − 05	24.77	EGR2
A_23_P96158	0.002943707	24.55	KRT17
A_23_P34915	0.001120758	23.39	ATF3
A_23_P71037	0.004629572	21.98	IL6
A_23_P46429	7.94*E* − 04	21.04	CYR61
A_24_P882732	0.036205057	19.08	
A_23_P97141	0.0211732	17.66	RGS1
A_23_P323751	9.21*E* − 05	17.26	FAM83D
A_33_P3316273	0.00369598	15.81	CCL3
A_23_P216225	0.0219925	15.72	EGR3
A_33_P3295203	0.001008955	15.65	HAS1
A_23_P131208	2.92*E* − 04	14.26	NR4A2
A_23_P214080	0.00224604	13.9	EGR1
A_33_P3214105	8.77*E* − 04	13.44	ATF3
A_33_P3390793	0.004063138	13.41	TRIM36
A_33_P3354607	0.001234311	13.09	CCL4
A_23_P79518	0.006241249	12.95	IL1B
A_23_P1331	0.001146301	11.08	COL13A1
A_23_P110569	7.10*E* − 04	10.2	TRIM36
A_23_P166408	0.003698398	10.04	OSM
A_32_P76627	1.51*E* − 04	10.02	
A_23_P207564	0.00151482	9.94	CCL4
A_33_P3299066	0.001036557	9.61	NR4A2
A_33_P3214393	0.008276591	9.56	
A_33_P3413741	0.032112285	9.53	OXTR
A_33_P3271594	0.001451045	9.49	TRIM54
A_24_P158089	0.003357552	8.93	SERPINE1

**Table 3:** Genes significantly downregulated in the remodeled vein compared to the control vein (top 30).

Probe name	FCAbsolute	*P* value	Gene symbol
A_23_P23783	18.99	0.009408315	MYOC
A_23_P121545	14.63	6.67*E* − 04	GPM6A
A_33_P3368193	10.96	2.33*E* − 05	PNLIPRP3
A_32_P92489	8.79	0.004908097	PKD1L2
A_24_P40626	8.15	0.011296479	GREM2
A_33_P3221408	8.14	0.004285622	NTNG1
A_23_P143526	7.15	0.004383178	S100B
A_23_P136777	7.14	3.89*E* − 04	APOD
A_23_P102331	7.10	0.003490915	SCN7A
A_33_P3421923	7.03	0.001119926	CADM3
A_23_P140384	7.00	0.026459113	CTSG
A_33_P3363799	6.94	0.002682039	NCAM1
A_24_P203134	6.80	0.024163503	DCAF12L1
A_24_P280684	6.69	0.03005707	FBXO40
A_23_P55544	6.51	0.004269639	CCBE1
A_23_P73571	6.45	0.03981546	MUM1L1
A_23_P212050	6.22	0.021448081	BCHE
A_33_P3336557	6.12	1.20*E* − 04	
A_23_P121676	6.07	0.014616995	CXXC4
A_23_P204885	6.01	0.007333652	PCDH20
A_23_P64919	5.92	0.012492463	RERGL
A_23_P422911	5.81	6.12*E* − 04	HS6ST3
A_23_P146233	5.79	0.01808302	LPL
A_23_P110624	5.76	0.003615111	CTNND2
A_23_P45185	5.69	0.00549277	FIGF
A_23_P110764	5.65	0.009343005	MYOT
A_23_P114862	5.41	0.039056532	ANGPTL7
A_23_P39251	5.31	9.92*E* − 04	PLIN5
A_23_P111402	5.28	0.008291814	RSPO3
A_33_P3400763	5.26	0.038730744	PLIN4

**Table 4:** Statistically overrepresented GO terms in the biological process category (*P* < 0.001).

Biological process						
GO accession (with AmiGO link)	GO term	Corrected *P* value	Count in selection	% count in selection	Count in total	% count in total
GO:0032502	Developmental process	2.100*E* − 11	77	29.8	3077	17.9
GO:0007275	Multicellular organismal development	1.340*E* − 10	67	26.0	2810	16.3
GO:0010033	Response to organic substance	3.530*E* − 10	40	15.5	698	4.1
GO:0001568	Blood vessel development	9.230*E* − 10	21	8.1	231	1.3
GO:0048514	Blood vessel morphogenesis	1.250*E* − 09	19	7.4	198	1.1
GO:0001944	Vasculature development	1.250*E* − 09	21	8.1	238	1.4
GO:0048545	Response to steroid hormone stimulus	1.360*E* − 09	23	8.9	183	1.1
GO:0001525	Angiogenesis	2.930*E* − 09	18	7.0	139	0.8
GO:0009653	Anatomical structure morphogenesis	4.930*E* − 09	30	11.6	1125	6.5
GO:0016265	Death	5.110*E* − 09	39	15.1	663	3.8
GO:0048646	Anatomical structure formation involved in morphogenesis	5.950*E* − 09	18	7.0	306	1.8
GO:0008219	Cell death	1.280*E* − 08	37	14.3	658	3.8
GO:0042221	Response to chemical stimulus	1.280*E* − 08	53	20.5	1264	7.3
GO:0048856	Anatomical structure development	1.320*E* − 08	42	16.3	2437	14.1
GO:0006950	Response to stress	2.080*E* − 08	51	19.8	1642	9.5
GO:0048731	System development	3.600*E* − 08	38	14.7	2284	13.3
GO:0032570	Response to progesterone stimulus	7.370*E* − 08	9	3.5	21	0.1
GO:0006915∣GO:0008632	Apoptosis	1.340*E* − 07	31	12.0	541	3.1
GO:0012501∣GO:0016244	Programmed cell death	1.900*E* − 07	31	12.0	549	3.2
GO:0042981	Regulation of apoptosis	2.470*E* − 07	35	13.6	796	4.6
GO:0032501∣GO:0050874	Multicellular organismal process	3.190*E* − 07	70	27.1	4154	24.1
GO:0043067∣GO:0043070	Regulation of programmed cell death	3.190*E* − 07	35	13.6	804	4.7
GO:0010941	Regulation of cell death	3.310*E* − 07	35	13.6	807	4.7
GO:0009887	Organ morphogenesis	3.510*E* − 07	26	10.1	685	4.0
GO:0009628	Response to abiotic stimulus	5.270*E* − 07	16	6.2	357	2.1
GO:0009725	Response to hormone stimulus	5.390*E* − 07	23	8.9	358	2.1
GO:0048519∣GO:0043118	Negative regulation of biological process	6.290*E* − 07	39	15.1	1756	10.2
GO:0009719	Response to endogenous stimulus	7.450*E* − 07	24	9.3	391	2.3
GO:0009605	Response to external stimulus	7.730*E* − 07	32	12.4	869	5.0
GO:0048513	Organ development	1.510*E* − 06	34	13.2	1682	9.8
GO:0009607	Response to biotic stimulus	2.160*E* − 06	23	8.9	385	2.2
GO:0070482	Response to oxygen levels	3.460*E* − 06	14	5.4	137	0.8
GO:0009266	Response to temperature stimulus	3.950*E* − 06	9	3.5	86	0.5
GO:0050896∣GO:0051869	Response to stimulus	6.040*E* − 06	89	34.5	3356	19.5
GO:0048523∣GO:0051243	Negative regulation of cellular process	8.220*E* − 06	38	14.7	1606	9.3
GO:0009408∣GO:0006951	Response to heat	8.220*E* − 06	9	3.5	61	0.4
GO:0006928	Cellular component movement	1.200*E* − 05	14	5.4	450	2.6
GO:0050793	Regulation of developmental process	1.590*E* − 05	8	3.1	670	3.9
GO:0048869	Cellular developmental process	1.600*E* − 05	24	9.3	1641	9.5
GO:0022603	Regulation of anatomical structure morphogenesis	2.530*E* − 05	5	1.9	228	1.3
GO:0051239	Regulation of multicellular organismal process	2.860*E* − 05	7	2.7	924	5.4
GO:0007565	Female pregnancy	3.100*E* − 05	13	5.0	104	0.6
GO:0030154	Cell differentiation	4.450*E* − 05	24	9.3	1576	9.1
GO:0042127	Regulation of cell proliferation	4.830*E* − 05	29	11.2	773	4.5
GO:0001666	Response to hypoxia	6.420*E* − 05	14	5.4	131	0.8
GO:0008284	Positive regulation of cell proliferation	7.720*E* − 05	16	6.2	410	2.4
GO:0048522∣GO:0051242	Positive regulation of cellular process	7.720*E* − 05	34	13.2	1806	10.5
GO:0051789	Response to protein stimulus	8.360*E* − 05	11	4.3	96	0.6
GO:0048518∣GO:0043119	Positive regulation of biological process	9.260*E* − 05	35	13.6	1982	11.5
GO:0043627	Response to estrogen stimulus	1.000*E* − 04	12	4.7	98	0.6
GO:0009991	Response to extracellular stimulus	1.000*E* − 04	6	2.3	204	1.2
GO:0042493∣GO:0017035	Response to drug	1.770*E* − 04	17	6.6	213	1.2
GO:0043066	Negative regulation of apoptosis	1.790*E* − 04	19	7.4	345	2.0
GO:0043069∣GO:0043072	Negative regulation of programmed cell death	2.200*E* − 04	19	7.4	350	2.0
GO:0051384	Response to glucocorticoid stimulus	2.200*E* − 04	10	3.9	70	0.4
GO:0050789∣GO:0050791	Regulation of biological process	2.440*E* − 04	109	42.2	7200	41.8
GO:0060548	Negative regulation of cell death	2.570*E* − 04	19	7.4	354	2.1
GO:0051707∣GO:0009613∣GO:0042828	Response to other organism	2.770*E* − 04	17	6.6	300	1.7
GO:0040011	Locomotion	2.800*E* − 04	16	6.2	415	2.4
GO:0009611∣GO:0002245	Response to wounding	2.800*E* − 04	21	8.1	507	2.9
GO:0031960	Response to corticosteroid stimulus	3.860*E* − 04	10	3.9	75	0.4
GO:0050794∣GO:0051244	Regulation of cellular process	4.070*E* − 04	108	41.9	6938	40.3
GO:0014070	Response to organic cyclic substance	4.200*E* − 04	12	4.7	114	0.7
GO:0051128	Regulation of cellular component organization	5.900*E* − 04	6	2.3	466	2.7
GO:0065007	Biological regulation	7.620*E* − 04	109	42.2	7592	44.1
GO:0051704∣GO:0051706	Multiorganism process	7.900*E* − 04	27	10.5	706	4.1
GO:0031099	Regeneration	8.770*E* − 04	6	2.3	65	0.4
GO:0007610	Behavior	9.680*E* − 04	12	4.7	449	2.6

**Table 5:** Statistically overrepresented GO terms in the molecular function category (*P* < 0.01).

Molecular function						
GO accession (with AmiGO link)	GO term	Corrected *P* value	Count in selection	% count in selection	Count in total	% count in total
GO:0005539	Glycosaminoglycan binding	9.960*E* − 06	14	5.4	149	0.9
GO:0005515∣GO:0045308	Protein binding	1.550*E* − 05	170	65.9	8104	47.0
GO:0001871	Pattern binding	3.120*E* − 05	14	5.4	164	1.0
GO:0030247	Polysaccharide binding	3.120*E* − 05	14	5.4	164	1.0
GO:0008201	Heparin binding	6.710*E* − 05	13	5.0	112	0.7
GO:0005126	Cytokine receptor binding	7.930*E* − 05	4	1.6	177	1.0
GO:0005125	Cytokine activity	2.200*E* − 04	12	4.7	193	1.1
GO:0005114	Type II transforming growth factor beta receptor binding	1.143*E* − 03	4	1.6	7	0.0
GO:0008083	Growth factor activity	2.365*E* − 03	13	5.0	160	0.9
GO:0005102	Receptor binding	3.106*E* − 03	24	9.3	873	5.1
GO:0030246	Carbohydrate binding	7.049*E* − 03	14	5.4	354	2.1

**Table 6:** Statistically overrepresented GO terms in the cellular component category (*P* < 0.01).

Cellular component						
GO accession (with AmiGO link)	GO term	Corrected *P* value	Count in selection	% count in selection	Count in total	% count in total
GO:0044421	Extracellular region part	2.190*E* − 09	49	19.0	937	5.4
GO:0031012	Extracellular matrix	7.420*E* − 07	25	9.7	339	2.0
GO:0005576	Extracellular region	3.880*E* − 06	69	26.7	1923	11.2
GO:0005615	Extracellular space	1.690*E* − 05	32	12.4	673	3.9
GO:0005578	Proteinaceous extracellular matrix	1.200*E* − 04	20	7.8	309	1.8
GO:0060205	Cytoplasmic membrane-bounded vesicle lumen	4.070*E* − 04	7	2.7	44	0.3
GO:0031983	Vesicle lumen	5.660*E* − 04	7	2.7	46	0.3
GO:0031093	Platelet alpha granule lumen	2.468*E* − 03	7	2.7	41	0.2

**Table 7:** Pathway analysis results.

Pathway name	LS_vs_control
Alpha6 beta4 integrin signaling pathway	0.793
Androgen receptor signaling pathway	0.528
Apoptosis mechanisms	0.124
B-cell receptor signaling pathway	0.023
G1 to S cell cycle control	1
Cell cycle	0.487
Delta-Notch signaling pathway	0.226
DNA replication	1
EGFR1 signaling pathway	0.856
FAS pathway and stress induction of HSP regulation	1
**Focal Adhesion**	**0.003**
G13 signaling pathway	0.269
G protein signaling pathways	0.258
Hedgehog signaling pathway	1
Apoptosis modulation by HSP70	1
Id signaling pathway	1
IL-1 signaling pathway	1
IL-2 signaling pathway	0.327
IL-3 signaling pathway	0.371
IL-4 signaling pathway	0.589
IL-5 signaling pathway	0.576
IL-6 signaling pathway	1
IL-7 signaling pathway	0.498
IL-9 signaling pathway	1
Human insulin signaling	0.387
Integrin-mediated cell adhesion	0.363
Kit receptor signaling pathway	0.051
MAPK cascade	1
MAPK signaling pathway	0.011
mRNA processing (*Homo sapiens*)	0.014
Notch signaling pathway	0.191
Ovarian infertility genes	1
p38 MAPK signaling pathway (BioCarta)	0.108
Regulation of actin cytoskeleton	0.834
Eukaryotic transcription initiation	0.511
Signal transduction of S1P	0.384
Signaling of hepatocyte growth factor receptor	1
T cell receptor signaling pathway	0.243
TGF-beta receptor signaling pathway	0.095
**TGF beta signaling pathway**	**0**
TNF-alpha/NF-*κ*B signaling pathway	0.752
Translation factors	0.368
Wnt signaling pathway	0.15
Wnt signaling pathway	0.051
Acetylcholine synthesis	1
Alanine and aspartate metabolism	—
Biogenic amine synthesis	1
Cholesterol biosynthesis	0.644
Eicosanoid synthesis	1
Electron transport chain	0.013
Fatty acid beta oxidation 1	0.403
Fatty acid beta oxidation 2	1
Fatty acid beta oxidation 3	1
Beta oxidation meta MAPP	0.264
Fatty acid omega oxidation	0.687
Fatty acid biosynthesis	0.426
Glucocorticoid and mineralcorticoid metabolism	1
Glutathione metabolism	0.399
Glycogen metabolism	0.261
Glycolysis and gluconeogenesis	0.235
Heme biosynthesis	1
TCA cycle	0.24
Mitochondrial LC-fatty acid beta-oxidation	0.635
Nuclear receptors in lipid metabolism and toxicity	0.389
Nucleotide metabolism	0.622
Pentose phosphate pathway	1
Prostaglandin synthesis and regulation	1
Statin pathway (PharmGKB)	1
Steroid biosynthesis	1
Synthesis and degradation of ketone bodies	1
Triacylglyceride synthesis	0.419
Tryptophan metabolism	0.501
Beta oxidation of unsaturated fatty acids	1
Urea cycle and metabolism of amino groups	—
GPCRs, class A rhodopsin-like	0.317
GPCRs, class B secretin-like	1
GPCRs, class C metabotropic glutamate, pheromone	1
GPCRs, other	1
Matrix metalloproteinases	0.652
Monoamine GPCRs	1
Nuclear receptors	1
Nucleotide GPCRs	1
Peptide GPCRs	0.081
Cytoplasmic ribosomal proteins	0.025
Small ligand GPCRs	1
ACE inhibitor pathway (*Homo sapiens*)	0.254
Adipogenesis human	0.101
Blood clotting cascade	0.155
Calcium regulation in the cardiac cell	0.431
Circadian exercise	0.754
Complement activation and classical pathway	0.646
Complement activation and classical pathway	0.022
**Cytokines and inflammatory response (BioCarta)**	**0**
**Hypertrophy model**	**0**
Inflammatory response pathway	0.054
Irinotecan pathway (*Homo sapiens*)	0.685
Oxidative stress	0.402
Proteasome degradation	0.278
**Myometrial relaxation and contraction pathways**	**0**
Striated muscle contraction	0.427
